# Inhalational monkeypox virus infection in cynomolgus macaques

**DOI:** 10.3389/fcimb.2012.00117

**Published:** 2012-09-17

**Authors:** Roy E. Barnewall, David A. Fisher, Ashley B. Robertson, Pauline A. Vales, Katherine A. Knostman, John E. Bigger

**Affiliations:** Battelle, ColumbusOH, USA

**Keywords:** monkeypox, inhalation, aerosol, exposure

## Abstract

An inhalation exposure system was characterized to deliver aerosolized monkeypox virus (MPXV), and a non-human primate (NHP) inhalation monkeypox model was developed in cynomolgus macaques. A head-only aerosol exposure system was characterized, and two sampling methods were evaluated: liquid impingement via an impinger and impaction via a gelatin filter. The aerosol concentrations obtained with the gelatin filter and impinger were virtually identical, indicating that either method is acceptable for sampling aerosols containing MPXV. The mass median aerodynamic diameter (MMAD) for individual aerosol tests in the aerosol system characterization and the NHP study ranged from 1.08 to 1.15 μm, indicating that the aerosol particles were of a sufficient size to reach the alveoli. Six cynomolgus macaques (four male and two female) were used on study. The animals were aerosol exposed with MPXV and received doses between 2.51 × 10^4^ to 9.28 × 10^5^ plaque forming units (PFUs) inhaled. Four of the six animals died or were euthanized due to their moribund conditions. Both animals that received the lowest exposure doses survived to the end of the observation period. The inhalation LD_50_ was determined to be approximately 7.8 × 10^4^ pfu inhaled. These data demonstrate that an inhalation MPXV infection model has been developed in the cynomolgus macaque with disease course and lethal dose similar to previously published data.

## Introduction

Smallpox, caused by the variola virus, a member of the *Poxviridae* family, is a highly contagious and debilitating systemic disease that is lethal to approximately 25% of its victims (Fenner et al., 1988, 1989). The World health Organization (WHO) initiated in 1959 a global operation to eradicate smallpox through vaccination, with the last case reported in 1977. Smallpox was officially considered eradicated in 1980 (Fenner et al., 1988; Henderson et al., 1999), but concerns over its use as a biological weapon have remained (Henderson et al., 1999). Control of a smallpox outbreak poses significant challenges to first responders and local and national health care systems. There is no FDA-approved treatment for smallpox beyond supportive care. The current international strategy for control of a smallpox outbreak calls for mass immunization of the general population with the vaccinia virus vaccine (CDC Smallpox Response Plan; Executive Summary, 2003). In preparation for this contingency, health care workers and many in the US military are currently being vaccinated. The conventional vaccine (ACAM2000) consists of a replicating vaccinia virus. Vaccinia vaccination was the cornerstone of the smallpox eradication campaign, and much of the world was vaccinated during that effort. However, serious complications are associated with vaccinia, particularly in immunocompromised individuals, where the vaccination can lead to serious complications including death (Lane et al., 1970; Thomas et al., 2008). To control an outbreak today, vaccination of at risk individuals would be enacted. However, there are certain groups or populations that vaccination is not recommended due to the risk of adverse reactions to vaccination (Fulginiti et al., 2003). Immunocompromised individuals cannot be vaccinated because uncontrolled replication of the vaccine may result in serious complications including death. The National Institute of Allergy and Infectious Diseases (NIAID) is directing a major effort to provide a safer vaccine, that may be offered to a population of individuals who otherwise would not be eligible to receive the ACAM2000 vaccine. To test the efficacy of these vaccines, animal models of disease must be developed.

Because smallpox is eradicated and ethical considerations, the efficacy of treatments and vaccines against smallpox cannot be determined in humans. Therefore, testing of smallpox therapeutics and new vaccines will require US FDA marketing approval according to 21 CFR 314 subpart I and 21 CFR 601 subpart H, known as the Animal Rule. To meet the Animal Rule, well-characterized animal models of smallpox are required, and a non-human primate (NHP) smallpox model likely will be required. Although the variola virus can infect NHPs, it does not result in a lethal systemic disease. Furthermore, the variola virus exists in only two repositories in the world and is not available for broad testing of therapeutics and vaccines.

Monkeypox virus (MPXV) is also a member of the Orthopoxvirus genus and presents a surrogate model for characterizing antivirals and vaccines to treat or prevent smallpox. Humans can be infected with MPXV, and the clinical presentation is similar to classic smallpox (Ladnyj et al., 1972; Parker et al., 2007; Chapman et al., 2010; Damon, 2011). Therefore, MPXV fulfills both criteria for a relevant smallpox surrogate: (1) it is a related virus that can infect an NHP and (2) it causes a lethal systemic infection.

When developing an animal model, it is important that the experimental route of infection be the same or similar to the anticipated route of infection in human disease. Transmission of smallpox occurs from aerosols from close contact with an infected individual. These infectious droplets can implant onto the nasal, oral, or pharyngeal mucous membranes or in the alveolar space of the lung and cause disease (Fenner et al., 1988). Therefore, infection is likely to occur anywhere along the respiratory tract. There have been several efforts to develop an NHP model of smallpox using the MPXV. One of the first well-described efforts involved the intravenous route of infection. This approach bypassed the early respiratory events, yet produced the severe systemic disease symptoms typically observed in human smallpox. This model has been used to test antiviral drug candidates (Huggins et al., 2009). Alternatives to the intravenous model have also been examined that include intratracheal, intranasal, and inhalation. The intratracheal method of exposing cynomolgus macaques to MPXV was recently described (Stittelaar et al., 2006; Goff et al., 2011). This approach attached a microsprayer to a bronchoscope for intratracheal infection. Kramski infected marmosets with cowpox virus that had been isolated from a lethal orthopox virus outbreak in New World monkeys, termed calpox. Calpox was lethal not only via the intravenous route but also by the intranasal route, and the marmosets reproducibly develop symptoms resembling smallpox in humans (Kramski et al., 2010). Small particle aerosols containing MPXV have been used to infect the lower respiratory tracts of cynomolgus macaques (Zaucha et al., 2001; Nalca et al., 2010) via an inhalational route. These studies were able to replicate the severe symptoms observed in intravenous infections, but required specialized equipment to generate aerosols containing MPXV.

In this manuscript, an aerosol exposure system was characterized for aersolizing MPXV and an inhalational infection of cynomolgus macaques with MPXV is described. The NHP exposure doses were performed with high precision relative to target dose. The disease presentation was shown to be representative of human smallpox. Respiratory symptoms and a prodromal period of infection prior to pox lesion development were observed and some but not all of the inhalation exposed animals developed pox lesions. These results corroborated the results obtained by previous investigators (Zaucha et al., 2001; Nalca et al., 2010).

## Materials and methods

### Animals

Six specific pathogen free *Macaca fascicularis* (Cynomolgus) monkeys (four male; two female) were obtained from a USDA certified vendor (Covance Labs, Alice, TX). The NHPs were free of obvious clinical signs of illness or malformations and tested negative for simian immunodeficiency virus (SIV), simian T-lymphotrophic virus 1 (STLV-1), macacacine herpesvirus 1 (herpes B virus), simian retrovirus 1 (SRV1) and SRV2, and for poxvirus antibodies prior to placement on study. The research was conducted in compliance with the Animal Welfare Act and followed the principles in the Guide for the Care and Use of Laboratory Animals from the National Research Council. Additionally, the research was conducted following an Institutional Animal Care and Use Committee (IACUC) approved protocol. The institution where the research was conducted is fully accredited by the Association for the Assessment and Accreditation of Laboratory Animal Care International (AAALAC). All MPXV exposed animals were housed within a Biosafety Level 3 (BSL-3) containment laboratory.

### Virus propagation and quantitation

MPXV (Zaire V79-I-005) strain was obtained from the Biodefense and Emerging Infections Research Resource Repository (BEI Resources). Monkeypox viral stocks were prepared in T-150 Vero cell flasks (Diagnostic Hybrids, Inc., Athens, OH). The virus was diluted in Eagle's minimal essential medium (EMEM, Fisher Scientific) with 5% fetal bovine serum (FBS, Fisher Scientific) without antibiotics to varying concentrations for use in a nebulizer for aerosol exposure. A virus plaque assay was used to quantitate the virus in the challenge material, impinger samples, and gelatin filter samples.

### Plaque assay

MPXV was enumerated in complete Eagles Minimum Essential Media (EMEM) with 0.1% gentamycin sulfate and 5% FBS and warmed to 37°C. Plaque assays were carried out on plates containing Vero 76 cells at ≥90% confluency obtained from Diagnostic Hybids Inc, (Anthens, OH). For each sample, one hundred mL of each dilution was added to each well of a twelve-well plate. Plates were incubated for 1 hour in at 37°C at 5% CO_2_, rocking plates approximately every 15 min. After one hour, one mL of complete media were added to each well and incubated for 4 days at 37°C. On day 3, media were removed from the plates and stained with 0.5–1 mL crystal violet solution for 15 min and rinsed with PBS. Once the cells were dry, the number of plaques were counted.

### Aerosol system characterization

The Battelle large animal aerosol exposure system was characterized for aerosolizing MPXV as determined via Spray Factor (SF) determination (aerosol conc/nebulizer conc). Three starting nebulizer concentrations (5.55 × 10^3^, 6.94 × 10^4^, and 7.19 × 10^5^ pfu/mL) were aerosolized in duplicate for 10 min. One day of testing was performed to characterize a head-only aerosol exposure system, and two sampling methods were evaluated. The aerosol system was operated as described previously (Galloway et al., 2004; Warren et al., 2011). The aerosol samples were collected by two methods head-to head in parallel: (1) into a glass impinger (Model 7541, Ace Glass Inc., Vineland, NJ) filled with approximately 20 mL of EMEM with one drop of anti-foam A (Dow Corning, Midland, MI) or (2) by impaction onto a 47 mm gelatin filter (Sartorius, Bohemia, NY). The aerosol particle size was determined during each test using an Aerodynamic Particle Sizer® (APS) spectrometer, model 3321 (TSI, Shoreview, MN). After sampling, each filter was dissolved into 10 mL of EMEM for quantification of MPVX. The impinger and gelatin filter samples were enumerated by the plaque assay method to quantify the MPXV counts per milliliter. The enumeration results, along with the volume used to dissolve the filter, sampling rate, sample volume, and sampling duration, were used in the calculation of the aerosol concentration expressed as plaque forming units (PFUs)/L of air (see section “Inhaled Dose Calculation”).

### Animal aerosol exposures

The Battelle large animal exposure system was used to expose animals individually, using a flow through design (Galloway et al., 2004; Warren et al., 2011). Four target exposure doses of 3.0 × 10^4^, 1.0 × 10^5^, 3.0 × 10^5^, and 9.0 × 10^5^ pfu of MPXV were delivered to the six monkeys. Briefly, each NHP was anesthetized by intramuscular injection of Telazol (tiletamine HCl and zolazepam HCl) at approximately 3 mg/kg. Each NHP was placed into a biological safety cabinet III (BSCIII) and loaded into a plethysmograph. Body plethysmography was performed real-time using Buxco Biosystems XA (Buxco Research Systems, Wilmington, NC) while the animal's head was placed within an exposure chamber. Air was supplied to the system by an in-house air system filtered through a carbon filter and two High Efficiency Particulate Air (HEPA) capsule filters. A modified Microbiological Research Establishment type three-jet Collison nebulizer (BGI, Waltham, MA) with a precious fluid jar was used to generate a controlled delivery of aerosolized MPXV from an EMEM suspension. The Collison nebulizers are designed to generate aerosols having an approximate mean diameter of 1–2 μm. Aerosol concentration was determined by analysis of atmospheric samples drawn from a port on the side of the exposure chamber. The samples were collected by two methods in parallel: (1) into a glass impinger (Model 7541, Ace Glass Inc., Vineland, NJ) filled with approximately 20 mL of media or (2) by impaction onto a 47 mm gelatin filter (Sartorius, Bohemia, NY). A vacuum pump pulled the sample into the impinger or filter at 6.0 ± 0.3 L/min. The virus impacted on the gelatin filter was recovered by dissolving the filter in 10 mL of EMEM. The filter samples were enumerated by the plaque assay method to quantify the MPXV counts per milliliter. The gelatin filter enumeration results, along with the volume used to dissolve the filter, sampling rate, sample volume, and sampling duration, were used in the calculation of the aerosol concentration expressed as pfu/L of air (see section “Inhaled Dose Calculation”).

### Plethysmography

Body plethysmography was performed real-time on each animal during agent exposure to measure important respiratory parameters. These parameters (tidal volume, total accumulated tidal volume or TATV, and minute volume) were calculated from the Buxco XA software by the measured volumetric displacement of air caused by the movement of the thoracic cavity of an animal while it was in the plethysmograph. The data generated for each animal was used to determine the TATV, which along with the aerosol concentration was used in calculating the inhaled dose. The plethysmograph was connected to a pneumotach (Hans Rudolph, Inc., Kansas City, MO) that was attached to a differential pressure transducer (Model DP-45; Validyne Engineering Corp., North Ridge, CA). Pressure differential measurements from inhalations and exhalations were transmitted to Biosystems XA software that calculated and recorded respiratory function. Prior to animal exposures, the plethysmography system was calibrated to establish unit (baseline) and air volume displacements from 5 to 40 mL to simulate animal respiration. This calibration was performed to encompass the respiration volume range of the animal model for accurate TATV measurements.

### Clinical observations and samples

Clinical observations of each NHP were recorded twice daily for 21 days post-exposure. Body temperatures were collected once daily from two transponders (shoulder and hip) beginning two days prior to exposure through the end of study. Body weights were collected on Days 0, 2, 4, 6, 10, 14, 18, and 21 days post-exposure. Blood and buccal swabs for virus quantification by qPCR were collected on Days 0, 2, 4, 6, 10, 14, 18, and 21 post-virus challenge. The blood was collected in EDTA tubes, 200–400 μL. Lesion counts were obtained on Days 0, 2, 4, 6, 10, 14, 18, and 21 post-exposure. The weights, temperature, and lesion counts were tabulated on a Clinical Assessment form on Days 0, 2, 4, 6, 10, 14, 18, and 21 to aid in clinical monitoring.

### Necropsy and histopathology

Necropsies were performed on all NHPs, and tissues collected for histology. Protocol-required tissue samples (brain, heart, lungs, and gross lesions) were placed in 10% neutral buffered formalin, processed to approximately 5-micron H&E-stained slides, and examined microscopically by an ACVP board-certified pathologist. Gross and microscopic diagnoses were entered into the PATH/TOX SYSTEM (Xybion Medical Systems Corporation) for data tabulation and analysis. Tissues from all NHPs were examined microscopically. Microscopic findings were graded semi-quantitatively according to the following scale, with the associated numerical score used to calculate average severity grades for each lesion by group and sex. Minimal (Grade 1) represented the least detectible lesion; mild (Grade 2) represented an easily discernible lesion; moderate (Grade 3) represented a change affecting a large area of the represented tissue; and marked (Grade 4) represented a lesion that approached maximal.

### Inhaled dose calculation

The total inhaled dose (*D*_inh_) was calculated from the gelatin filter sample concentration *(C)*, sampling parameters, total inhaled tidal volume (*V*_inh_) and exposure duration *(T)* according to the equation below. The total number of MPXV captured during each exposure was the product of the gelatin filter concentration (*C*_*i*_) and the volume the filter was dissolved in (*C* × *V*). The total number of PFUs was divided by the amount that was sampled through the filter during the exposure time (*S* × *T*). The aerosol concentration was (*C* × *V*) (*S* × *T*)^−1^. The inhaled dose was the product of the aerosol concentration multiplied by the TATV measured during plethysmography as detailed in Equation 1:

$$D_{\text{inh}}=\left[\left(C\times V_{i}\right){\left(S\times T\right)}^{-1}\right]\times \left(V_{\text{inh}}\right)=C_{a}\times V_{\text{inh}}$$

*D*_inh_ = Total inhaled dose (PFU)

*C* = Filter sample concentration (PFU/mL)

*V*_*i*_ = Filter sample volume (mL)

*S* = Filter sampling rate (L/min)

*T* = Exposure time (min)

*V*_inh_ = Total inhaled tidal volume (L), as determined by plethysmography

*C*_*a*_ = Aerosol concentration (PFU/L)

### Statistical analysis

Statistical analysis for the LD_50_ for the inhaled dose data was fitted to a logistic regression model to survival probability as a function of base-10 logarithm of the measured aerosol dose. Since the two lowest dose animals survived, and the four higher dose animals succumbed, a conventional logistic regression model such as Spearman-Karber would provide an indeterminate result. Thus, a logistic regression procedure that used a penalized maximum likelihood (Firth, 1993) was implemented in Stata 11.1 using the “firthlogit” command. The model fit to the data was this approximate equation:

$$\text{In}[\text{Probability}\;\text{of}\;\text{Survival}/(1-\text{Probability}\;\text{of}\;\text{Survival})]\;=-17.27+3.53\times \log_{10}(\text{Measured}\;\text{Dose})$$

### Nucleic acid isolation and quantitative polymerase chain reaction (qPCR)

#### Nucleic acid isolation

Lung, brain, and heart samples from each animal were homogenized in 1 mL of phosphate buffered saline (PBS). Total nucleic acid was isolated from whole blood samples (100 μL) using the Specific B protocol on a NucliSens® easyMAG™ instrument (BioMérieux, l′Etoile, France). The homogenate was then poured through a 40 μM cell strainer, with the homogenizer and cell strainer rinsed with a total of 2 mL of NucliSens easyMAG Lysis Buffer. Buccal swabs were placed in a 1 mL solution consisting of 97% PBS, 2% FBS, and 1% Penicillin/Streptomycin. The entire recoverable volume of the solution was then added to 2 mL of NucliSens Lysis Buffer. Whole blood was collected into ETDA tubes, with 100 μL taken and added to 2 mL of NucliSens Lysis Buffer. Total nucleic acid was then isolated from these lysates using the NucliSens easyMAG. In brief, easyMAG Magnetic Silica is added to each sample after loading the lysate into sample vessels in the instrument. The Magnetic Silica then binds total nucleic acid during a 10 min incubation at room temperature, and the Magnetic Silica is then collected, and subsequently washed twice with NucliSens easyMAG Extraction Buffer 1, then twice with NucliSens easyMAG Extraction Buffer 2, and once with NucliSens easyMAG Extraction Buffer 3, with a final elution of total nucleic acid in 50 μL of NucliSens easyMAG Extraction Buffer 3.

Total nucleic acid was isolated from whole blood samples, throat swabs and homogenized tissues using the Specific B protocol on a NucliSens® easyMAG™ instrument (BioMérieux) according to manufacturer's methods.

#### Quantitative Real-Time PCR (qRT-PCR)

Total purified nucleic acid was eluted in a final volume of 40 μL. Each nucleic acid sample was assayed by qRT-PCR for the MPXV N3R gene in duplicate on a 7900HT real-time PCR system (Applied Biosystems, Life Technologies Corp., Carlsbad, CA). Each 25 μL reaction contained 5 μL of sample with the remaining volume consisting of TaqMan® Gene Expression Master Mix (2X, Applied Biosystems), sterile water, and a custom gene expression assay (Applied Biosystems) consisting of previously developed primers and a 3′-minor groove binding probe specific for a portion of the MPXV N3R gene (Kulesh et al., 2004). The final concentration of primers and probe were 900 nM and 250 nM, respectively. Thermal cycling on the 7900HT instrument was as follows: 50°C for 2 min, 95°C for 10 min, followed by 45 cycles of 95°C for 15 sec and 60°C for 1 min. A plasmid containing a cloned insert of the primer and probe specific portion of the N3R gene (Retrogen, Inc.) was run as a 10-fold serial dilution (2 × 10^7^ to 2 copies/μL) reference standard curve with all samples to determine the concentration of gene copies in each sample. The final concentration for each sample was reported as copies per milliliter of original sample. The sequence of the primers, probe, and amplicon are as follows:

Forward Primer: AACAACCGTCCTACAATTAAACAACA

Reverse Primer: CGCTATCGAACCATTTTTGTAGTCT

Probe: 6FAM-TATAACGGCGAAGAATATACT-NFQ

Amplicon: AACAACCGTCCTACAATTAAACAACAttactttTATAACGGCGAAGAATATACTgaaattgatagatcgaaaaaagccactaataaaaacagttggttaattacttcaggctttAGACTACAAAAATGGTTCGATAGCG

## Results

### Aerosol system characterization

**Figure 1:**
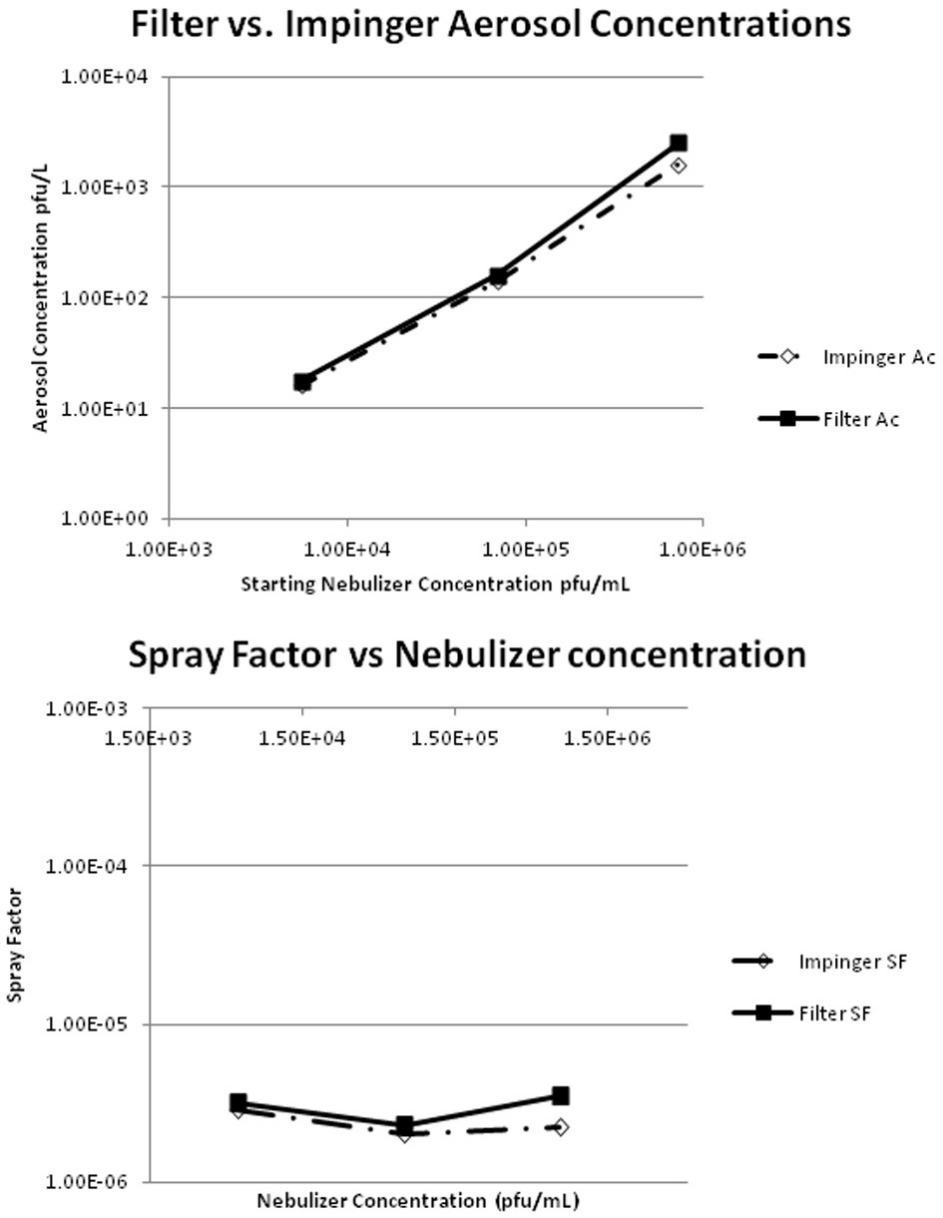
**Shows the results of the monkeypox virus aerosol concentrations (Ac) for the gelatin filter compared to the impingers.** It also shows the Spray Factor (SF) results for the gelatin filter compared to the impinger. The aerosol concentrations were equivalent by impingement and gel filtration capture, they were proportional to the starting nebulizer concentration, and there was no difference in the Spray Factor Values between the gelatin filter and impinger.

The virus aerosol was characterized by conducting two 10-min sprays at three concentrations. Figure 1 shows the comparisons of the impinger and gelatin filter samplings of the aerosol chamber during these characterization experiments. The aerosol concentrations obtained with the filter compared to the impinger were virtually identical, indicating that either method is acceptable for sampling aerosols containing MPXV (Figure 1). Figure 1 indicates that there was a linear increase in the aerosol concentration as the starting nebulizer concentration was increased. Additionally, Figure 1 shows that the SF was relatively constant among the nebulizer concentrations tested, with a mean value of 2.4 × 10^−6^ for the impinger and 3.0 × 10^−6^ for the gelatin filter. As a result, gelatin filter results were used in the animal dose calculations. The aerosol contained particles with mass median aerodynamic diameter (MMAD) of 1.10 μm and ranged from 1.08–1.15 μm for each individual test, and the geometric standard deviation (GSD) was 1.61 μm with a range of 1.58–1.64 μm for each individual test. These results indicated that the aerosol distribution was relatively monodispersed and contained particles capable of reaching the deep lung in animal testing (Schlesinger, 1985).

The SF can be used as a fundamental indicator to measure and assess the performance of the aerosol system over time (i.e., when operating the aerosol system under the same conditions, mean SF should be the same or fall within the same range time after time). The SF is the ratio of the concentration of agent in the aerosol (aerosol concentration) to the starting concentration used in the aerosol generator. As a consequence, the SF value is a unitless factor that represents the dilution that is to be expected during an aerosol experiment using the same bio-agent in the same aerosol system under the same conditions. This enables the assessment of the performance of the aerosol exposure process and whether it is stable (the same) for each individual test or exposure within a test day and across multiple test days.

### Aerosol exposures of nHPs

Following the aerosol characterization, the aerosol system was used to expose six cynomolgus macques to nebulized MPXV. The actual inhaled exposure dose was calculated for each animal by multiplying the actual inhaled air volume of the animal while in the aerosol chamber (as measured by realtime plythesmography) against the aerosol chamber's virus concentration as measured by gelatin filtration. Table 1 is a summary of the target doses compared to the actual exposure dose received by each NHP along with the day post-exposure the animal died or was euthanized. The actual exposure doses were well within the assay confidence of the targeted doses. The aerosol contained particles with MMAD of 1.07 μm and ranged from 1.06–1.09 μm for each individual test and the GSD was 1.58 with a range of 1.56–1.70 for each individual test.

**Table 1:** **Exposure dose summary**.

**Animal ID**	**Sex**	**Weight (kg)**	**Targeted exposure dose (pfu)**	**Actual exposure dose (pfu)**	Day of death or euthanasia^*^
C0412403	Male	8.0	3.00 × 10^4^	4.25 × 10^4^	−
C0501065	Male	8.7	3.00 × 10^4^	2.51 × 10^4^	−
A08407	Female	2.5	1.00 × 10^5^	1.22 × 10^5^	8
C0506081	Male	9.1	1.00 × 10^5^	2.85 × 10^5^	9^*^
A09166	Female	3.0	3.00 × 10^5^	3.90 × 10^5^	7
C0404035	Male	10.1	9.00 × 10^5^	9.28 × 10^5^	10

* Animal found dead; all others euthanized/euthanized moribund.

### Clinical findings

Table 2 is a summary of the cutaneous lesions that developed on the animals during the post-exposure observation period. Lesion presentation began 6 days following exposure, and then only on the two highest dosed animals. Animal A08407 succumbed without apparent lesion presentation. Animal C0404035 was the only animal to present with severe lesions. Animal A08407 died 8 Days post-exposure, and at necropsy and histopathology presented with pneumonia and intrunuclear inclusion bodies consistent with pox infection. Death was likely from pneumonia before secondary viremia and associated skin lesions.

**Table 2:** **Lesion count summary**.

**Animal ID**	**Actual exposure dose (pfu)**	**Lesion count (arms, legs, ventral, head)^a^**					
		**Day 0**	**Day 2**	**Day 4**	**Day 6**	**Day 8**	**Day 10**
C0412403	4.25 × 10^4^	0	0	0	0	0	1, 1, 1, 1
C0501065	2.51 × 10^4^	0	0	0	0	0	1, 1, 1, 1
A08407	1.22 × 10^5^	0	0	0	0	0	–
C0506081	2.85 × 10^5^	0	0	0	0	0	(1, 1, 1, 1)^b^
A09166	3.90 × 10^5^	0	0	0	0, 1, 1, 0	–	–
C0404035	9.28 × 10^5^	0	0	0	1, 2, 2, 2	1, 3, 3, 3	3, 3, 3, 3

a Lesion count : 1, <25 lesions; 2, 25–99 lesions; 3, >99 lesions.

b Observation taken on Day 9 at euthanasia.

Virus burden in this study was measured by qPCR quantification of virus from tissues taken at necropsy and by qPCR quantification of virus in buccal swabs and whole blood taken every other day during the first 10 days after exposure. Low levels of virus were detected in buccal samples on Day 4 in one animal and in the blood of four animals. By Day 6 all animals had detectable levels in the blood and four out of six had detectable levels in buccal samples (Table 3), suggesting that detection of virus in saliva was dependent upon hematogenous spread of virus and most likely from the lungs. In the two animals that survived to end of study, virus was detected at extremely low levels, if at all, in blood and saliva after Day 14.

**Table 3:** **Summary of blood, buccal, and tissue qPCR results**.

**Animal ID**	**Day 0**	**Day 2**	**Day 4**	**Day 6**	**Day 8**	**Day 10**	**Day 14**	**Day 18**	**Day 21**
**BLOOD**									
C0412403	LLOQ	LLOQ	5.1 × 10^3^	7.0 × 10^4^	1.5 × 10^5^	4.0 × 10^5^	3.1 × 10^5^	1.2 × 10^3^	LLOQ
C0501065	LLOQ	LLOQ	LLOQ	7.4 × 10^4^	3.0 × 10^5^	2.6 × 10^5^	4.1 × 10^4^	LLOQ	LLOQ
A08407	LLOQ	LLOQ	7.8 × 10^3^	1.8 × 10^5^	2.7 × 10^5^	NA	NA	NA	NA
C0506081	LLOQ	LLOQ	LLOQ	9.1 × 10^4^	3.6 × 10^5^	NA	NA	NA	NA
A09166	LLOQ	LLOQ	9.3 × 10^3^	4.8 × 10^5^	NA	NA	NA	NA	NA
C0404035	LLOQ	LLOQ	2.5 × 10^4^	6.9 × 10^5^	4.5 × 10^6^	4.8 × 10^6^	NA	NA	NA
**BUCCAL SWABS**									
C0412403	LLOQ	LLOQ	LLOQ	2.5 × 10^3^	1.1 × 10^5^	2.5 × 10^5^	1.1 × 10^5^	6.3 × 10^3^	8.5 × 10^2^
C0501065	LLOQ	LLOQ	LLOQ	LLOQ	1.4 × 10^5^	4.1 × 10^5^	6.9 × 10^4^	3.5 × 10^3^	3.7 × 10^3^
A08407	LLOQ	LLOQ	LLOQ	LLOQ	3.0 × 10^4^	NA	NA	NA	NA
C0506081	LLOQ	LLOQ	LLOQ	8.4 × 10^2^	2.1 × 10^5^	NA	NA	NA	NA
A09166	LLOQ	LLOQ	3.2 × 10^2^	9.0 × 10^3^	NA	NA	NA	NA	NA
C0404035	LLOQ	LLOQ	5.3 × 10^3^	5.8 × 10^4^	1.9 × 10^6^	4.6 × 10^6^	NA	NA	NA
**Animal ID**		**Lung**		**Brain**		**Heart**		**Day of death**	
C0412403		LLOQ		LLOQ		3.7 × 10^2^		21	
C0501065		6.5 × 10^6^		LLOQ		LLOQ		21	
A08407		1.8 × 10^6^		LLOQ		4.0 × 10^3^		8	
C0506081		3.5 × 10^4^		4.3 × 10^2^		4.1 × 10^3^		9	
A09166		2.2 × 10^8^		1.3 × 10^3^		2.8 × 10^4^		7	
C0404035		1.2 × 10^8^		3.2 × 10^3^		2.1 × 10^4^		10	

NA, not applicable; LLOQ, below lower limit of quantification (200 copies/g tissue; 1000 copies/mL blood, 200 copies/mL swab). Results reported as genomes/mL or genomes/g tissue.

Dose dependent virus shedding in saliva or viremia was not readily apparent, though the animal that received the highest exposure dose (C0404035) did appear to have higher blood and saliva virus titers than any others on any given day.

As expected, the highest virus titers were found in lung tissues at necropsy (Table 3) as this was the site of dosing and initial infection. Interestingly, Animal C0501065, which was still showing clinical signs of illness, survived and was recovering at the end of in-life testing on Day 21 post-exposure. The animal no longer presented with virus in its blood and saliva but still showed 6 logs of virus genome in its lungs Day 21 post-infection. This is in contrast to animal C041203, which had no detectable virus in its lung, and only minimal amounts in its heart.

### Gross necropsy and histopathology

Both monkeys in the targeted 3 × 10^4^ pfu/animal group survived to the study end on Day 21 post-exposure. These animals had grossly evident skin pocks on multiple body sites but no internal gross lesions. All four monkeys exposed to higher concentrations of MPXV died or became moribund and were euthanized between study Days 7–10. Lung discoloration, bronchial lymph node enlargement, and/or skin pocks were evident in three of the four monkeys, while one lacked any gross lesions. All gross lesions noted were typical of monkeypox.

All monkeys that succumbed or became moribund during the study had microscopic lesions consistent with monkeypox. Lesions included surface (skin) and respiratory epithelial necrosis and reactive epithelial hyperplasia, with some epithelial cells in both sites containing eosinophilic to amphophilic intranuclear and/or intracytoplasmic inclusion bodies (virus inclusions). Fibrinosuppurative inflammation, edema, hemorrhage, and lymphoid necrosis were evident in the skin, tongue, lungs, and/or bronchial lymph nodes. Specifically, lung lesions had a prominent bronchial/peribronchial airway pattern (bronchopneumonia) indicative of the inhalation route of infection, as well as a slightly more subtle perivascular to interstitial pattern, consistent with a septicemic phase.

### Statistical analysis

**Figure 2:**
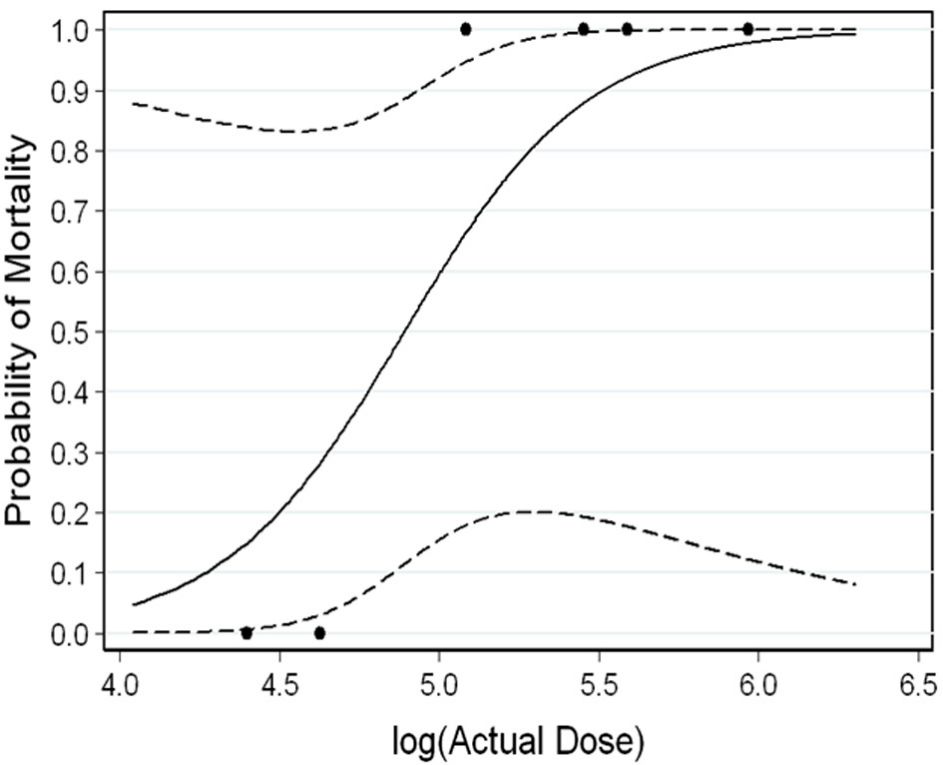
**Survival Data Logistic Regression Curve showing the survival data as well as the logistic regression curve fit to the data.** Dashed lines represent the 95% confidence intervals.

The median lethal dose (LD_50_) and LD_90_ were estimated to be 77,900 pfu and 326,000 pfu, respectively. These results are similar to those reported in the recent literature (Nalca et al., 2010). The logistic regression model did not have a statistically significant slope (*p* = 0.18), so 95% Fieller's confidence intervals for these percentiles are not bounded. The reason the 95% confidence intervals are not bounded is due to the small number of animals tested and because the two animals that received the lowest doses survived and the four higher doses animals died. Figure 2 is a plot showing the survival data as well as the logistic regression curve fit to the data.

## Discussion

Infection of pox virus by different exposure routes will lead to variations in the course of disease in NHPs and humans (Hahon, 1961; Reynolds et al., 2006; Chapman et al., 2010). The inhalation route of infection is preferred in an animal model as this closely resembles the natural route of infection in human smallpox (Hahon, 1961; Henderson et al., 1999). As a result, the inhalation MPXV model in the cynomolgus macaque was developed and characterized.

The initial step was the characterization of MPXV in the inhalation exposure system. The aerosol exposure system characterization testing indicated that the aerosol concentrations determined by impingement and gel filtration capture were equivalent and were proportional to the starting nebulizer concentration. Also, there was no difference in the SF values between the gelatin filter and impinger. The results from the characterization testing were used to determine the starting concentration needed in the nebulizer to achieve the desired target doses to the NHPs by use of the SF.

The doses delivered to the NHPs were very close to the desired target doses, indicating that the aerosol exposure system performed identically to the characterization day, and suggest low system variability both within-a-day and between-days. The difference in the SF value between impingers and gelatin filters was 20% or lower at the nebulizer concentrations tested and is well within the 0.5 log variability of plaque assay enumeration method, indicating that the values are essentially the same. Although the one day of SF characterization testing produced excellent results, ideally at least three days of characterization testing is recommended to be performed along with a larger number of replicates at each concentration tested. This would enable the determination of a more accurate and reliable with-a-day and between-day variability so that the variability of doses delivered to an animal could be more accurately predicted in future animal testing.

Accounts from other facilities have shown that NHPs that succumb to lethal aerosolized MPXV do so via severe respiratory disease (Zaucha et al., 2001; Chapman et al., 2010), and recently some are reported without presentation of typical pox skin lesions (Nalca et al., 2010). This observation was also noted in the current study and was reflected in the presentation of disease and death of three of the four NHPs that succumbed on this study. Animals C0506081 and A09166 died with few pox lesions, and A08407 had no pox lesions evident. This is in contrast to the intravenous lethal exposure model where the NHP succumbs to severe systemic monkeypox disease with total body coverage of lesions (Chapman et al., 2010).

The NHPs succumbed to infection in a dose-dependent fashion, with the lowest dose group surviving exposure. The animals succumbed due to respiratory disease, as reflected by the amount of virus recovered from the lungs, as compared to other organs (see Table 3) and the lesions in the lung.

The mortality data suggest that weight and/or NHP sex may be a contributing factor to this infection model. Two males, weight ≥8 kg, survived exposure with 3 × 10^4^ pfu monkeypox. Further, the two other males (weight ≥9 kg) that received 9 × 10^5^ pfu and 1 × 10^5^ pfu survived longer than the two females (weight ≥3 kg). This question can only be resolved by conducting a well controlled age/weight/sex comparison study. To mitigate this potential affect, special consideration should be given to the size, age and sex of animals placed on future studies.

Virus in blood and buccal samples was detected as early as day 4 post-aerosol challenge and peak between days 6 and 10, which corroborates what has been observed by other investigators (Nalca et al., 2010).

Multiple objectives were fulfilled from these tests. An aerosol exposure system was characterized using aerosolized MPXV, and it was shown that gelatin filters and impingers of type model 7541 generated equivalent enumeration results when sampling the MPXV aerosol. Following characterization of the aerosol exposure system, an NHP inhalation study determined a lethal exposure dose of MPXV, and an inhalation cynomolgus macaque model of MPXV infection was developed. Actual NHP exposures were performed with high precision relative to target dose, and the lesions and lethality were similar to the previously published data for the cynomolgus monkey (Zaucha et al., 2001, Nalca et al., 2010).

### Conflict of interest statement

The authors declare that the research was conducted in the absence of any commercial or financial relationships that could be construed as a potential conflict of interest.
